# Inequality trends of health workforce in different stages of medical system reform (1985-2011) in China

**DOI:** 10.1186/s12960-015-0089-0

**Published:** 2015-12-08

**Authors:** Kaiyuan Zhou, Xinyi Zhang, Yi Ding, Duolao Wang, Zhou Lu, Min Yu

**Affiliations:** Department of Aerospace Health Services, Fourth Military Medical University, Xi’an, Shaanxi Province China; Department of Otolaryngology, Xi’an Children Hospital, Xi’an, Shaanxi Province China; Department of Pharmacy, Xijing Hospital, Fourth Military Medical University, Xi’an, Shaanxi Province China; Department of Clinical Sciences, Liverpool School of Tropical Medicine, Pembroke Place, Liverpool, L3 5QA UK; Department of Health Services, Fourth Military Medical University, Xi’an, Shaanxi Province China; Department of Health Services, Academy of Military Medical Sciences, Beijing, China

**Keywords:** Inequality, Health workforce, Medical system reform

## Abstract

**Introduction:**

The aim of this study was to identify whether policies in different stages of medical system reform had been effective in decreasing inequalities and increasing the density of health workers in rural areas in China between 1985 and 2011.

**Methods:**

With data from China Health Statistics Yearbooks from 2004 to 2012, we measured the Gini coefficient and the Theil L index across the urban and rural areas from 1985 to 2011 to investigate changes in inequalities in the distributions of health workers, doctors, and nurses by states, regions, and urban-rural stratum and account for the sources of inequalities.

**Results:**

We found that the overall inequalities in the distribution of health workers decreased to the lowest in 2000, then increased gently until 2011. Nurses were the most unequally distributed between urban-rural districts among health workers. Most of the overall inequalities in the distribution of health workers across regions were due to inequalities within the rural-urban stratum.

**Discussions and conclusions:**

Different policies and interventions in different stages would result in important changes in inequality in the distribution of the health workforce. It was also influenced by other system reforms, like the urbanization, education, and employment reforms in China. The results are useful for the Chinese government to decide how to narrow the gap of the health workforce and meet its citizens’ health needs to the maximum extent.

## Introduction

The quantity and distribution of the health workforce, the most important aspect of health care systems, directly influenced the quality and quantity of health services and the long-term development of the medical system [1,2]. In China, the distribution of the health workforce had changed a lot through medical system reform. In the 1970s of the last century, China’s barefoot doctor system was regarded as a successful example of solving shortages of medical services in developing countries [3]. Then, China was ranked 101st out of 191 countries in health distribution in the World Health Report 2000 [4]. The Chinese government admitted that the previous medical system reform was a “failure” in 2005. It led to health inequality and poor accessibility of health services [5]. Since it shifted from a planned economy to market economy in 1978, China has obtained great substantial economic achievement. However, what happened to the Chinese health workforce in the periods of medical system reform?

Medical system reform started in China in 1985. As the fundamental change of political and economic circumstances, it has gone through four stages [6]: First, from 1985 to 1992, China began the market-oriented medical system reform, which encouraged hospitals to liberalize charges of medical treatment. Consequently, public hospitals began to have the for-profit tendency to alleviate the tension of having much less funding from the state [7]. Second, from 1992 to 2000, the medical system reform went through further marketization, which began to influence the public welfare of health services, and finally resulted in the low availability and high costs of health services for the commons [8]. At the end of this stage, the government formally stopped the policy of assigning jobs for undergraduates [9], and a large-scale expansion of college enrollment began. Third, from 2000 to 2005, the drawbacks of market reform arose intensively. The government and specialists began to rethink the market-oriented reform profoundly, and SARS in 2003 made the central government push forward the community health services [10]. In this stage, the government issued many intervention policies to attract health workers to rural areas. Four, from 2005 till now, a report by the State Council Development Research Center pointed out that over the past 10 years, medical system reform was a “failure” in 2005. Then, a new round of medical system reform began in 2006 [5]. The government began to focus on health service quality of medical institutions for public welfare, and the government generally implemented intervention policies attracting health workers to rural areas. These policies also were constantly revised and updated. The government issued distinctive policies in accordance with the reform guidance in each stage. An inappropriate medical policy would inevitably result in long-term adverse effects, such as health talent loss and decrease in public welfare [11].

Many other factors also could result in the inequality of the health workforce. Zhang Qiang analyzed different kinds of factors and concluded that government policies in the health workforce and economic conditions among regions were the vital factors, which prevented human resources flowing into the grassroots health institutions in China [12]. And regions with a better socio-economic environment would attract more health workers, including better living conditions, access to education for children, and availability of employment for spouses [13,14]. How the policies in the different stages of the medical system reform impacted on the inequality of the health workforce in China—that was the main question this article wanted to answer.

Many scholars demonstrated that the health inequality in China had been reflected in the disparities between the urban and rural areas and between different regions [15-17]. Some Chinese research studies in inequality of the health workforce were cross-sectional studies [18-23]. Anand measured the inequalities of distribution of the health workforce in 1990, 2000, and 2005, all in the middle stage of the medical system reform [24]. But few longitudinal studies and quantitative analyses focused on inequalities of the health workforce at different stages of medical system reform. As for the measurement of inequality, Anand introduced the use of the Gini coefficient and Theil index to measure the fairness of the distribution of human resources for health and applied these methods to China and India [24,25]. Sousa used these methods to measure the changes in the inequalities in the distribution of the health workforce in Brazil [26]. Pallikadavath also used them to measure the health workforce of India’s public health care system [27].

In this article, we chose the Gini coefficient and Theil L index to measure inequalities of the health workforce at the urban-rural level in different stages of the medical system reform. We then evaluated the tendency of these inequalities over different periods and accounted for the sources of the inequalities by Theil L decomposition. Then, we could identify whether policies in the different periods had a positive effect on inequalities. This study offered policymakers a distinct and important reference. It may be beneficial to make more targeted and informed policies to resolve the problem of the lack of health workers in remote rural areas.

## Data and methods

We used the data on China’s health workforce from China Health Statistics Yearbooks [28] and population and economic data from China Statistics Yearbooks, all in the Chinese language. These two kinds of yearbooks were published by the Chinese central government. The data in them were powerful and reliable. Health workers include doctors, nurses, pharmacists, technicians, and other technical staff. Doctors are those who pass a licensing examination and are registered at a county or higher level health authority as either licensed doctors or licensed assistant doctors. Nurses are those who have obtained nursing certification with an associate degree (3 years tertiary nursing education) or higher or graduates from secondary education programs with a diploma (2 years nursing education after high school) and recommended by a health authority at the provincial level or above. Village doctors, called “barefoot doctors” before 1985, are those who have not obtained the qualifications of licensed doctors or licensed assistant doctors, registered at a county health authority, and engaged in prevention, health care, and general medical services in the village clinics. Data of village doctors usually list separately in the health yearbooks. The doctor and nurse, the basic constitution of the health worker, were chosen to assess the inequality of the health worker in the urban-rural stratum. Data in different stages of the medical system reform could be obtained. As the data were often updated in the newest yearbook, we chose the most updated data when they were not consistent in different yearbooks.

The population of urban and rural areas was represented by the population of cities and counties until 2009, and the health workers of cities and counties did not include the village doctors until 2008. As for the official classification of the urban-rural stratum, the health worker was classified by urban and rural groups from 1949 to 1984, by city and county groups from 1985 to 2004, then by urban and rural groups again after 2005. The urban area includes the direct-controlled cities’ district and prefecture-level cities’ district, and the rural area contains counties and county-level city. As to common regional distinction in China, the East includes 11 provinces or direct-controlled cities, which are Beijing, Tianjin, Hebei, Liaoning, Shanghai, Jiangsu, Zhejiang, Fujian, Shandong, and Hainan; the Midland contains 8 provinces, which are Shanxi, Jilin, Heilongjiang, Anhui, Jiangxi, Henan, Hubei, and Hunan; and the West covers 12 provinces or autonomous regions or direct-controlled cities, which are Inner Mongolia, Chongqing, Guangxi, Sichuan, Guizhou, Yunnan, Xizang, Shaanxi, Gansu, Qinghai, Ningxia, and Xinjiang [28].

The Gini coefficient and Theil L index were chosen to investigate the inequality trends in the densities of health workers, doctors, and nurses in the different stages of the medical system reform in China. Both of them were identified by Anand [25] to measure inequalities in the distribution of health workers and account for the sources of the inequalities in a country. If *h*_*i*_ = number of health workers in a geographical unit *i* and *p*_*i*_ = number of people in the unit *i*, then the health worker density in unit *i* is *x*_*i*_ = *h*_*i*_/*p*_*i*_. If the total number of health workers in the country is *H* = *h*_1_ + *h*_2_ + … + *h*_*n*_ = ∑_*i*_ *h*_*i*_ and the total number of people (population) in the country is *P* = *p*_1_ + *p*_2_ + … + *p*_*n*_ = ∑_*i*_ *p*_*i*_, then the national health worker density is *X* = *H*/*P*; Theil L = ∑_*i*_ (*p*_*i*_/*P*) log (*X*/*x*_*i*_) = ∑_*i*_ (*p*_*i*_/*P*) [log (*X*) − log (*x*_*i*_)]. Let the cumulative proportion of the population up to unit *i* be *F*_*i*_ = ∑_*k* = 1_^*i*^ (*p*_*i*_/*P*) and the cumulative proportion of health workers up to unit *i* be *Φ*_*i*_ = ∑_*k* = 1_^*i*^ (*h*_*i*_/*H*), *F*_0_ = *Φ*_0_ = 0, then the Gini coefficient is *G* = 1 − ∑_*i* = 0_^*n* − 1^ (*F*_*i* + 1_ − *F*_*i*_) (*Φ*_*i* + 1_ + *Φ*_*i*_).

The Gini coefficient and Theil L index both took the values between 0 and 1, with higher values indicating higher levels of inequality [29]. However, the Gini coefficient, a well-known measure to account for inequalities in the economical field, had four levels for its value: below 0.3 was the best state of average, between 0.3 and 0.4 normal, beyond 0.4 warning, and reaching 0.6 or more perilous state of the highly unfair [30]. But it could not be decomposed to explain the sources of the inequalities. The Theil L index could be decomposed to explain within-group inequality and between-group inequality, which was more appropriate to interpret the sources of inequalities. The between-group component of the Theil L index measured inequality due solely to variations in health worker density across urban-rural strata. And the within-group component of the Theil L index measured inter-unit (East-Midland-West strata) inequality within urban or rural groups. Due to data limits, we just used the Gini coefficient and Theil L index without decomposition to analyze the inequality trends of health workforce density in the different stages in China. As supplementary, we also used the decomposed Theil L index to explain the sources of inequalities from 2003 to 2011. All analyses above were performed by Excel 2007.

## Results

### Inequality changes of urban-rural health workforce in the different stages in China

As shown in Table 1, the density of health workers per thousand people consistently increased from 3.28 in 1985 to 4.58 in 2011, with just a slight fluctuation in 2005. More specifically, it decreased gradually from 7.92 in 1985 to 5.17 in 2005, then rose slowly to 7.9 in 2011 in urban districts. By contrast, it grew steadily from 2.09 in 1985 to 3.19 in 2011 in rural districts but a pause in 2007. When the health workers in rural districts contained village doctors, rural health workers per thousand people rose steadily from 3.61 in 1985 to 4.04 in 2000, a decrease of 3.92 in 2005, and then steadily increased to 4.91 in 2011; national health workers per thousand people fell from 4.69 to 4.3 in 1995 and then steadily increased to 6.44 in 2011. Second, doctors per thousand persons grew slowly from 1.36 in 1985 to 1.85 in 2011. More precisely, it fell from 3.35 in 1985 to 2.31 in 2000, and then ascended steadily to 3 in 2011 in urban districts, while it went up gradually from 0.85 in 1985 to 1.33 in 2011 in rural districts. Finally, nurses per thousand people nationwide grew from 0.61 in 1985 to 1.66 in 2011. Specifically, it fluctuated from 1.85 in 1985 to 1.59 in 1995 and afterwards went up steadily to 3.29 in 2011 in urban districts, whereas it consistently increased from 0.3 in 1985 to 0.98 in 2011.

**Table 1:** **Change of health workforce per thousand people from 1985 to 2011**

**Stage**	**Year**	**Health worker**			**Doctor**			**Nurse**			**Health worker (including village doctor)**		
		**Urban**	**Rural**	**Total**	**Urban**	**Rural**	**Total**	**Urban**	**Rural**	**Total**	**Urban**	**Rural**	**Total**
Stage 1 (1985–1992)	1985	7.92	2.09	3.28	3.35	0.85	1.36	1.85	0.30	0.61	7.92	3.61	4.69
	1990	6.59	2.15	3.45	2.95	0.98	1.56	1.91	0.43	0.86	6.59	3.61	4.40
Stage 2 (1992–2000)	1995	5.36	2.32	3.59	2.39	1.07	1.62	1.59	0.49	0.95	5.36	3.87	4.30
	1999	5.24	2.38	3.64	2.33	1.14	1.67	1.64	0.52	1.02	5.24	4.00	4.43
Stage 3 (2000–2005)	2000	5.17	2.41	3.63	2.31	1.17	1.68	1.64	0.54	1.02	5.17	4.04	4.45
	2003	4.84	2.19	3.42	2.08	0.97	1.48	1.59	0.50	1.00	4.84	3.32	3.94
Stage 4 (2005–now)	2005	5.82	2.69	3.57	2.46	1.26	1.60	2.10	0.65	1.06	5.82	3.92	4.74
	2007	6.44	2.69	3.76	2.61	1.23	1.62	2.42	0.70	1.19	6.44	3.99	5.12
	2009	7.15	2.94	4.15	2.83	1.31	1.75	2.82	0.81	1.39	7.15	4.47	5.76
	2011	7.90	3.19	4.58	3.00	1.33	1.82	3.29	0.98	1.66	7.90	4.91	6.44

Note: data were obtained from China Health Statistics Yearbooks

Table 2 presented that the urban-rural Gini coefficient of health workers fell from 0.304 in 1985 to 0.187 in 2000, after that, went up to 0.21 in 2007, then stabilized at that level. By contrast, the Gini coefficient of health workers including village doctors fell from 0.163 in 1985 to 0.059 in 2005, then rose to 0.119 in 2007, and stabilized until 2011. And the Theil L index had similar trends to the corresponding Gini index. Then, the urban-rural Gini coefficient of doctors dropped from 0.313 in 1985 to 0.166 in 2000 and afterwards rose steadily to 0.191 in 2011, whereas the Theil L index similarly fell from 0.204 in 1985 to 0.056 in 2000 and, after that, increased steadily to 0.08 in 2011. Lastly, the urban-rural Gini coefficient of nurses declined from 0.42 in 1985 to 0.271 in 2000, then ascended steadily to 0.287 in 2007, and then decreased gradually to 0.267 in 2011, while the Theil L index similarly fell from 0.385 in 1985 to 0.15 in 2000, afterwards rose steadily to 0.186 in 2007, and then declined gently to 0.171 in 2011.

**Table 2:** **Gini coefficient and Theil L index of** **urban-rural** **health workforce from 1985 to 2011**

	**Year**	**Health worker**		**Doctor**		**Nurse**		**Health worker (including village doctor)**	
		**Gini**	**Theil L**	**Gini**	**Theil L**	**Gini**	**Theil L**	**Gini**	**Theil L**
Stage 1 (1985–1992)	1985	0.304	0.192	0.313	0.204	0.420	0.368	0.163	0.059
	1990	0.260	0.139	0.255	0.135	0.350	0.253	0.131	0.038
Stage 2 (1992–2000)	1995	0.196	0.079	0.187	0.073	0.280	0.160	0.071	0.011
	1999	0.192	0.075	0.174	0.061	0.279	0.160	0.064	0.009
Stage 3 (2000–2005)	2000	0.187	0.071	0.166	0.056	0.271	0.150	0.059	0.007
	2003	0.196	0.078	0.188	0.072	0.279	0.164	0.093	0.017
Stage 4 (2005–now)	2005	0.190	0.074	0.166	0.056	0.279	0.168	0.098	0.019
	2007	0.211	0.094	0.184	0.070	0.287	0.186	0.119	0.028
	2009	0.211	0.096	0.186	0.073	0.282	0.185	0.116	0.028
	2011	0.210	0.097	0.191	0.080	0.267	0.171	0.116	0.028

Note: data were obtained from China Health Statistics Yearbooks

### Inequality source of health workforce distribution in China

The data above in 1985-2011 was directly grouped by statistics yearbook in urban and rural areas. Due to lack of data for specific regions, we used the data between 2003 and 2011, which could be used to calculate the decomposition of the Theil L index, and test the above results. However, these two sector data were different in statistical caliber. The urban-rural stratum of the East, Midland, and West regions was stratified by the city and county in statistics. In view of Chinese conditions, most of the cities were developed areas, and most of the counties were developing or undeveloped areas. But there were more health workers in the county’s developed areas, which would increase the number of health workers in rural districts and then result in bias. Even so, it could reflect the real situation to some degree. In addition, the East, Midland, and West regions meant the developed, developing, and undeveloped areas in China, respectively.

As Table 3 shows, the Theil L index between urban-rural health workers rose from 0.087 in 2003 to 0.111 in 2007 and then gradually declined; similarly, the Theil L index between urban-rural doctors increased from 0.074 in 2003 to 0.095 in 2007 and then gradually accelerating declined; the Theil L index between urban-rural nurses rose from 0.1625 in 2003 to 0.185 in 2007, stabilized to 2009, and then dropped sharply. All three trends were the same as the results of the last sector. The Theil L index between groups (urban and rural areas) contributed more than 95% of the Theil L index.

**Table 3:** **Gini coefficient and Theil L index of health workforce from 2003 to 2011**

	**Health worker**				**Doctor**				**Nurse**			
**Year**	**Gini**	**Theil L**	**Between group** **(% of overall)**	**Within group** **(% of overall)**	**Gini**	**Theil L**	**Between group** **(% of overall)**	**Within group** **(% of overall)**	**Gini**	**Theil L**	**Between group** **(% of overall)**	**Within group** **(% of overall)**
2003	0.211	0.087	0.086 (98.85)	0.001 (1.15)	0.197	0.074	0.073 (98.65)	0.001 (1.35)	0.277	0.163	0.1617 (99.51)	0.0008 (0.49)
2005	0.223	0.097	0.095 (97.94)	0.002 (2.06)	0.206	0.081	0.080 (98.77)	0.001 (1.23)	0.285	0.169	0.167 (98.82)	0.002 (1.18)
2007	0.241	0.111	0.109 (98.20)	0.002 (1.80)	0.223	0.095	0.094 (98.95)	0.001 (1.05)	0.299	0.185	0.182 (98.38)	0.003 (1.62)
2009	0.235	0.106	0.104 (98.11)	0.002 (1.89)	0.207	0.081	0.080 (98.77)	0.001 (1.23)	0.298	0.185	0.183 (98.92)	0.002 (1.08)
2011	0.108	0.019	0.003 (15.79)	0.016 (84.21)	0.103	0.018	0.0001 (0.57)	0.0174 (99.43)	0.144	0.040	0.023 (57.50)	0.017 (42.50)

Note: data were obtained from China Health Statistics Yearbooks

## Discussions and conclusions

**Figure 1:**
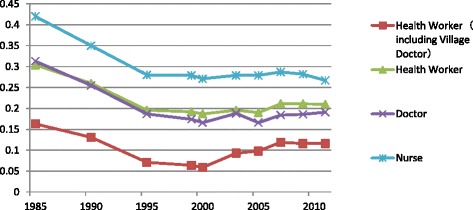
Gini coefficient of urban-rural health workforce from 1985 to 2011

The results showed that there were great improvements in increasing the availability of health workers in China from 1985 to 2011. The overall inequalities in the distribution of health workers decreased to the bottom at 2000, then increased gently until 2011 (as Figure 1 shows). The inequalities in the distribution of doctors, nurses, and health workers (including village doctors) have the same trend. Policies in the different stages had a similar impact on the inequalities of each type of health worker distribution. Then, we took a “health worker,” for example, to discuss the inequality trend in the following part.

The nurse was the kind of health worker which was more unequally distributed between urban-rural districts. The inequality degree of doctors was similar with health workers. According to the China Health Statistics Yearbook 2012, the ratio of doctor to nurse was 1:0.91 in 2011, lower than the lowest Chinese standard of 1:2, much lower than the average of developed countries [31]. The whole country still lacked a huge number of nurses. Then, nurses had more options to choose employers and stay in cities. After including village doctors, the inequality of the health worker distribution decreased obviously. Village doctors could provide basic medical care for rural citizens, although they might not have a high educational degree and medical ability. They played an important role in improving the accessibility of health services for peasants in China.

We found that the source of the inequalities was mainly from the urban-rural structure by Theil L decomposition. This was consistent with Hao Chan’s study, who found that the health inequality was mainly from the urban-rural disparity [32]. Under the urban-rural dual economic system, villages lagged far behind cities in income, consumption, health care, and educational fields. According to Dewen Wang’s study, the gap was bigger after the reform, and the consumption level in rural areas was the same with the urban level 16 years ago [33]. Reducing the urban-rural disparity was the fundamental method to solve the urban-rural inequality of health worker distribution.

The overall inequalities in the distribution of health workers decreased to the bottom at the end of the second stage of the medical system reform (in 2000). During the same time, the availability of health services decreased seriously due to the series of medical market reforms [15]. It was contradictory. Then, we checked the original data [28], and we found that the urban health worker from 1985 to 2000 increased by 19.42%, while the rural increased by 15.43%. The urban population increased by 82.94%, whereas the rural increased by only 0.09% during the same period. The ratio of urban health worker to rural rose from 1.18 in 1985 to 1.22 in 2000. The Chinese government fully promoted the urbanization process from 1985 to 2000 [34]. The growth of the health worker could not meet the vast health needs of the fast and fiercely increasing population in the urban areas. The improvement of the fairness of health worker distribution was not due to the improvement in the country but due to the reduction in the urban areas. The inequality of health worker distribution was influenced not only by health policy implementation but by the whole country development.

From the beginning of the third stage of the medical system reform, the inequalities in the distribution of health workers increased gently until 2011, as a negative effect of continued medical market reform. National market reform changed people’s mind. They attached great importance to money more than ever [35]. Medical market reform turned hospitals into profit-oriented institutions [36]. And health workers would like to be in major hospitals in cities. The situation became worse when human resources were gradually configured through the market. Government began to formally cancel the policy of assigning jobs for undergraduates in 1996, fully stopping in 2000 [9]. Meanwhile, a large-scale expansion of college enrollment began, and the growth rate even reached 47.4% in 1999 [37]. The number of students admitted by medical universities and colleges (including doctoral and graduate students, undergraduates, and college students) was 108 384 in 1999, an increase of 44.15% than before. After that, it maintained rapid growth till now [28]. The massive increasing medical graduates found jobs through the talent market and concentrated in large cities and large medical institutions. The urban health worker grew dramatically, an increase of 129.94% from 2000 to 2011, while the rural increased slowly, an increase of only 7.51% during the same period.

In the fourth stage of the medical system reform, the inequalities in the distribution of health workers were stable, with tiny fluctuations. That was the profit from a new round of public-welfare-oriented medical system reform, officially launched in 2007. It provided the Urban Employee Basic Medical Insurance (UEBMI), Urban Residents Basic Medical Insurance (URBMI), and New Rural Cooperative Medical Care (NRCMC) to cover all residents in China, enhanced the three-level health security system, and prompted the development of primary health care organizations through reimbursement limitations. Take URBMI in Xi’an city, for example, URBMI reimbursed 60% of medical expenditures in the primary grade hospitals and 40% of medical expenditures in the top grade hospitals [38]. And the reimbursement starting point of URBMI was 250 RMB in community health services, 350 RMB in primary grade hospitals, 500 RMB in middle grade hospitals, and 700 RMB in top grade hospitals [38]. All these measures were formulated to encourage patients to go to primary grade hospitals. Then, the grassroots health organizations would develop positively, and health workers were willing to go and stay in them.

The government issued and implemented many intervention policies to attract health workers to rural areas from the third stage. Many studies showed that rural recruitment and training, financial and professional incentives, and regulatory and administrative measures were effective interventions [39,40]. In China, further measures by the central government were implemented to prompt health workers to flow towards rural areas, such as the “Physician Supporting Rural Health Project” and free orientation training for rural general practitioners [41]. The local government also made many supporting policies. In Shaanxi province, the “Zhenxing Program for Rural Grassroots Personnel Team” was carried out from 2006. This program included the health workers below the level of township hospital. It provided rural personnel at the grassroots level with an annually special fund of 37 500 000 RMB which covered program bounty, fellowship, academic education, continuing education, and so on. The participants were allowed to attend Civil Service Exam and given preference for employment after 3 years service in the program. They also were given a 1 incremental-point grade within the salary scale. These interventions alleviated the inequality situation. But they were still not enough. Based on the principles of economics, the greater the number of health workers the more health workers were willing to work in rural areas. In fact, China still lacked a number of health workers as a whole. Then, it would increase the inequality of urban-rural health worker distribution. There may be two reasons for that: one was that the number of medical students was not enough; another was that the bad medical environment made medical graduates switch to another job [42,43].

This study has highlighted some critical issues in terms of the geographical distribution of skilled health workers through the new evaluation methods. Geographical areas with more health workers were more likely to have better population health, for the quantity of health workers was related to the availability of health services, especially in remote and rural areas. Although China’s health worker density increased year by year, especially for doctors, of which density had exceeded the world average (1.42 per thousand people) [44], the ratio of doctor to nurse was still low, which suggested the shortage of nurses and the inefficiency of the health system [45]. However, this type of analysis was essential for the government to decide how to meet its citizens’ health needs to the maximum extent.

We also should take into consideration the limitations of the data in this study. We used the health worker density and population data directly from the statistics yearbook, which could only evaluate the quantity of urban and rural health workers at the national level, not enough to calculate the decomposition of the Theil L index. However, we could use these data to identify the changes of inequality of health workers in urban-rural stratum in different periods in China. To overcome the problem above, we found health worker data of each province distributed by urban-rural stratum from the statistics yearbook in 2004-2012. With these data, we explored the source of inequality through the decomposition of the Theil L index. Although the two kinds of data were from various datasets, we also could get doubtless results. Because the trends calculated from these two datasets were consistent, and each comparison of urban-rural stratum was from the same data. And the results in this study were in line with recent studies, which showed great improvement year by year but unbalanced region development in the equality of distribution of health workers in China [46]. Such as it was, there were some biases in detailed numerical value. Given the expansion of investment in the poorest regions, West and remote rural areas in recent years, with strategies such as the western talent training plan [47] to increase the quality and quantity of health workers and increase the availability of health services, further analyses using the methods presented in this paper will be vital to monitor the effects of these policies to reduce inequalities in the distribution of health workers at the urban-rural stratum.
